# Genome sequence of the stress-tolerant yeast *Debaryomyces hansenii* DSM 3428

**DOI:** 10.1128/mra.00181-26

**Published:** 2026-05-06

**Authors:** Gabriele Ellena, Simon Draguet, Surya Gupta, Irina Spacova, Natalie Leys, Sarah Lebeer, Felice Mastroleo

**Affiliations:** 1 Microbiology Unit, Nuclear Medical Applications, Belgian Nuclear Research Center (SCK CEN), Mol, Belgium; 2 Laboratory of Applied Microbiology and Biotechnology (LAMB), Department of Bioscience Engineering, University of Antwerp, Antwerp, Belgium; 3 Faculty of Sciences, Université libre de Bruxelles (ULB), Brussels, Belgium; 4 U-MaMi Centre of Excellence, University of Antwerp 26660, Antwerp, Belgium; University of Manitoba, Winnipeg, Manitoba, Canada

**Keywords:** yeasts, genome organization, biotechnology

## Abstract

We present the complete genome sequence of *Debaryomyces hansenii* DSM 3428, assembled using long- and short-read sequencing data. This genome provides a valuable reference for investigating the genetic basis of stress tolerance and biotechnologically relevant traits in this yeast.

## ANNOUNCEMENT

*Debaryomyces hansenii* is a halotolerant yeast in the family Saccharomycetaceae, of interest for food-related applications (1), for astrobiological (2) studies, and space biotechnological (3, 4) applications in life-support systems. Here, we report the genome sequence of *Debaryomyces hansenii* DSM 3428; this genome provides a reference resource for comparative and functional genomic studies of this species.

The strain was obtained from the German Collection of Microorganisms and Cell Cultures (accession no. DSM 3428) and was originally isolated from spoiled sake (country unknown). No additional sampling or isolation procedures were performed in this study.

The strain was cultivated in liquid yeast extract–peptone–dextrose medium (5–7) at 25°C under aerobic conditions (180 rpm). Cells were harvested by centrifugation, washed, and resuspended in 500 µL of Zymo 1× DNA/RNA Shield prior to submission to Plasmidsaurus Inc. (Eugene, OR, USA). Genomic DNA extraction was performed by Plasmidsaurus using the Zymo Quick-DNA Miniprep Plus Kit (Zymo Research, Irvine, CA, USA) with Zymolyase pretreatment (Zymo Research). Library preparation and sequencing were performed by Plasmidsaurus using a hybrid approach combining Oxford Nanopore and Illumina platforms, following their standard workflow (https://plasmidsaurus.com/technical-documentation/genome). Oxford Nanopore libraries were prepared using v14 library preparation chemistry (Oxford Nanopore Technologies) with an amplification-free, tagmentation-based protocol, resulting in minimal fragmentation of genomic DNA. No additional size-selection step was applied prior to sequencing.

Long-read sequencing was performed on an Oxford Nanopore PromethION P24 using R10.4.1 flow cells, generating 259,756 reads totaling 1,434,080,893 bp, corresponding to 1.4 Gb of data (~120× genome coverage), with a maximum read length of 126,292 bp. Read quality and length distributions were assessed using NanoPlot (1.46.2). Low-quality long reads were filtered using Filtlong (v0.3.1), and reads were subsampled to ~100× coverage to optimize assembly performance. *De novo* genome assembly was carried out using Flye (2.9.6-b1802) with an estimated genome size of 12 Mb (Table 1).

**TABLE 1:** Software tools, versions, and parameters used for sequencing quality control, genome assembly, polishing, and annotation^*a*^

Software	Version	Purpose	Parameters
NanoPlot	v1.46.2	Quality control of Nanopore long reads (length, quality, coverage)	--fastq --only-report
FastQC	v0.12.1	Assessment of sequencing quality metrics (GC, adapters, base quality)	--nano
Filtlong	v0.3.1	Filtering low-quality Nanopore reads prior to assembly	Default parameters
SeqKit	v2.12.0	Subsampling Nanopore reads to reduce excessive coverage	sample -s 42 -p 0.88
Flye	v2.9.6-b1802	*De novo* genome assembly from Nanopore long reads	--nano-raw --genome-size 12m—iterations 3
Minimap2	v2.30-r1287	Alignment of Nanopore reads to draft assemblies	-x map-ont
Racon	v1.5.0	Long-read consensus polishing of draft assemblies	Default parameters, two rounds
Medaka	v*2.2.0*	Neural network-based polishing of Nanopore assemblies	Default model selection
BWA-MEM2	v2.2.1	Alignment of Illumina short reads to assembled genome	Default parameters
Polypolish	v0.6.1	Illumina-based polishing to correct single-nucleotide polymorphisms and small indels	Default parameters
QUAST	v5.3.0	Assembly quality assessment (contiguity, GC, N50)	Default parameters
BUSCO	v6.0.0	Genome completeness assessment	Lineage: saccharomycetes_odb10, default parameters
Funannotate	v1.8.17	Eukaryotic genome annotation pipeline	Default parameters
Augustus	v3.5.0	Ab initio gene prediction	Species model: *Debaryomyces hansenii*

^*a*^ Default parameters were used unless otherwise specified.

The draft assembly was polished with two rounds of Racon (1.5.0) using Nanopore reads aligned with minimap2 (2.30-r1287), followed by Medaka (2.2.0) polishing to correct systematic Nanopore sequencing errors.

Illumina paired-end sequencing (2 × 150 bp) was performed on a NextSeq 2000 platform, producing 82,134,172 reads used for polishing of the long-read assembly. Short reads were quality controlled using fastp with quality filtering (Phred ≥15, length ≥50 bp, adapter trimming, and poly-G trimming) prior to alignment to the long-read assembly. Illumina paired-end reads were subsequently mapped to the assembly using BWA-MEM2 (2.2.1), and Polypolish (0.6.1) was applied to correct residual single-nucleotide polymorphisms and small indels.

Assembly quality was assessed using QUAST (v5.3.0), and completeness was evaluated using BUSCO (6.0.0) with the saccharomycetes_odb10 data set. Genome annotation was performed using Funannotate (v1.8.17), including genome cleaning, repeat masking, contig sorting, ab initio gene prediction, and functional annotation.

The final genome assembly comprised 46 contigs with a total length of 11.40 Mb, an N50 of 1.07 Mb, and a GC content of 36.9%. No ambiguous bases (Ns) were present in the assembly. BUSCO analysis indicated 99.5% completeness (99.2% single copy and 0.3% duplicated), with no fragmented genes detected, demonstrating a near-complete and high-quality fungal genome assembly. Genome annotation predicted 5,804 protein-coding genes.

## Data Availability

This Whole Genome Shotgun project has been deposited in DDBJ/ENA/GenBank under accession no. JBUDIF000000000. The version described in this paper is the first version, JBUDIF010000000. Raw sequencing reads have been deposited in the NCBI Sequence Read Archive under BioProject accession no. PRJNA1417632. Data are publicly available via the following links: SRX32515305 (Illumina), and SRX32515304 (Nanopore).
